# Psilocybin in Neuropsychiatric Disorders: Seeking Valuable Evidence in History, Pure Science, Clinical Trials and Real-World Data (RWD)

**DOI:** 10.3390/brainsci16040358

**Published:** 2026-03-26

**Authors:** Piotr Skalski, Katarzyna Pękacka-Falkowska, Agnieszka Pluto-Prądzyńska, Michał K. Owecki

**Affiliations:** 1 Department of the History and Philosophy of Medical Sciences, Poznan University of Medical Sciences, 60-356 Poznan, Poland; kpekacka@ihpan.edu.pl (K.P.-F.);; 2 Tadeusz Manteuffel Institute of History, Polish Academy of Sciences, 00-272 Warszawa, Poland; 3 Department of Immunology, Chair of Pathomorphology and Clinical Immunology, Poznan University of Medical Sciences, 60-806 Poznan, Poland; agapp@ump.edu.pl

**Keywords:** psilocybin, psilocybin-assisted therapy (PAT), real-world data (RWD), evidence, psychopharmacology

## Abstract

**Highlights:**

**What are the main findings?**

• Psilocybin demonstrates consistent antidepressant, anxiolytic, and anti-addictive effects, yet its therapeutic outcomes depend not only on pharmacology but also on contextual factors such as set and setting, therapeutic support, and patient expectations.
• Standard randomized controlled trials (RCT) show major limitations when applied to psilocybin: difficulties with placebo design, compromised blinding, highly selective participant samples, and reductionist methodologies that fail to capture the pharmacological–psychological interaction inherent in psychedelic therapy.

**What are the implications of the main findings?**

• The evaluation of psilocybin requires research frameworks beyond traditional RCTs, particularly through the integration of real-world evidence (RWE), longitudinal observational data, and qualitative approaches that better reflect natural clinical environments and diverse patient populations.
• Establishing reliable conclusions about psilocybin’s efficacy and safety will require large-scale, multisite, long-term studies involving heterogeneous groups, as well as methodological models that recognize psilocybin-assisted therapy (PAT) as a complex intervention combining pharmacology and structured psychotherapy.

**Abstract:**

**Background/Objectives:** Psilocybin has re-emerged as a promising intervention for neuropsychiatric disorders including major depressive disorder, treatment-resistant depression, anxiety associated with life-threatening illness, obsessive compulsive disorder, and substance use disorders. However, conventional randomized controlled trials (RCTs)—the current gold standard in evidence-based medicine—may not adequately capture the therapeutic complexity of psilocybin, which depends not only on pharmacological action but also on contextual, psychological, and interpersonal factors. This critical narrative review aimed to evaluate the adequacy of existing clinical research frameworks for assessing psilocybin’s therapeutic potential and to explore alternative methodologies that may better reflect real-world clinical conditions. **Methods:** Using the Web of Science Core Collection database, we identified and analysed the ten most cited clinical studies on psilocybin published between 2015 and 2025 inclusive. Additional literature was included through reference cross-checking, systematic reviews, meta-analyses, and interdisciplinary sources covering neurobiology, history, and real-world evidence (RWE). The review synthesizes clinical outcomes, methodological constraints, and epistemic considerations relevant to psychedelic-assisted therapy. **Results:** Evidence from highly cited trials demonstrates rapid and sustained antidepressant and anxiolytic effects of psilocybin, with notable benefits also observed in addiction treatment. However, significant methodological limitations were identified, including selection bias, challenges in placebo design and blinding, small sample sizes, and the underrepresentation of diverse populations. Psilocybin outcomes were strongly influenced by subjective experience and contextual factors such as set and setting. Emerging RWE studies revealed heterogeneous patterns of response and provided insights unattainable through RCTs alone. **Conclusions:** Psilocybin shows considerable therapeutic promise, but current RCT methodologies capture only part of its clinical effects. Comprehensive evaluation will require larger and more diverse clinical trials, long-term follow-up, standardized psychotherapeutic protocols, and the integration of RWE to reflect real-world practice. Psychedelic-assisted therapy should be conceptualized as a complex intervention that combines pharmacological and psychotherapeutic components.

## 1. Introduction

The development of efficient therapies for neuropsychiatric disorders is a key challenge for both clinical psychiatry and pharmacology. Major depressive disorder (MDD) is among the most frequently diagnosed mental disorders, a leading cause of suicide attempts, often resulting in disability or death. Globally, depression affects an estimated 350 million people, while nearly 9% of adults in the United States experience a depressive episode annually [1,2]. Depression is just the tip of the iceberg, as commonly diagnosed conditions also include obsessive–compulsive disorder (OCD), posttraumatic stress disorder (PTSD), and anxiety associated with life-threatening illness.

Despite significant progress in understanding the aetiology of neuropsychiatric disorders, current treatment methods remain far from satisfactory [3]. Currently available pharmacotherapies are efficient in approximately 50% of patients with MDD receiving long-term treatment [4]. Furthermore, repeated treatment failures can worsen patients’ clinical outcomes and discourage continued treatment adherence [1]. The slow pace of psychopharmacological development, along with criticism aimed at this subfield of medical science, is forcing researchers and investors to change their approach in the search for novel and more efficient pharmacological treatments that will meet the expectations of doctors and patients [5,6].

Against this backdrop, the recent renewed interest in classic psychedelics (e.g., psilocybin, LSD, MDMA) comes as no surprise. Unconventional approaches such as psilocybin-assisted therapy (PAT) and microdosing are being explored, particularly for patients unresponsive to standard modalities [7]. Current evidence suggests that psilocybin is safe under controlled clinical conditions. Promising results from numerous clinical studies also indicate its therapeutic 8. However, psilocybin remains under investigation and is only legal in certain countries for therapeutic, clinical trial, or pilot programs. In 2020, Oregon became the first U.S. state to legalise the therapeutic use of psilocybin through Measure 109. Two years later, Colorado passed Proposition 122, legalising psilocybin for both recreational and therapeutic use. In mid-2023, Australia became the first country to legalise psilocybin for depression treatment at the national level [8]. Phase III trials of psilocybin are also underway [ClinicalTrials.gov ID: NCT05624268], which could lead to its approval as a prescription medication in other countries [9]. In several other countries in Europe and Latin America, the consumption of certain psychedelic substances is permitted for recreational purposes [10]. In addition, there are reports of the potential use of psilocybin in neurological and neuropsychiatric diseases, including Alzheimer’s disease [11], chronic migraine [12], substance use disorders [13], and as part of comprehensive palliative therapy [14].

Evidence-based medicine (EBM) has shaped contemporary standards for evaluating the efficacy and safety of therapies, emphasizing a hierarchy of scientific evidence. At the top of this hierarchy are randomized controlled trials (RCTs), which are widely regarded as the most reliable method for assessing medical interventions. RCTs have become the “gold standard” of clinical research because of their ability to minimize bias. This is achieved through key methodological principles, such as ensuring an equal distribution of confounding factors between the study and control groups, eliminating subjective assessments, and controlling for variables. However, the unique characteristics of psilocybin call into question the universal applicability of standard RCTs and pose challenges for evaluating the real therapeutic value of this substance.

## 2. Materials and Methods

This study was designed as a critical narrative review rather than a systematic review. Its aim was not to synthesise all available clinical evidence on psilocybin but to analyse the methodological frameworks that have shaped contemporary research on PAT. We sought to identify what is currently known, what remains uncertain, and what cannot yet be reliably inferred regarding the therapeutic potential of psilocybin from clinical, historical, and philosophical perspectives. The review critically assesses the adequacy of current evidence-generation methods and proposes alternative research directions that may allow for a more comprehensive understanding of psilocybin-based therapies.

### 2.1. Main Questions

Psilocybin differs from conventional psychotropic medication in that its therapeutic effects are strongly influenced by subjective experience, psychological state/expectations of the individual and the external conditions (set and setting), as well as the therapeutic relationship. This raises a central methodological question: Can RCTs adequately capture the full complexity of psilocybin’s therapeutic action?

While RCTs aim to isolate pharmacological effects, psilocybin outcomes depend on contextual, experiential, and psychological factors that are difficult to standardise. Consequently, alternative methodologies—such as real-world data (RWD) studies, longitudinal designs, and qualitative approaches—ds may offer important complementary insights, capturing long-term efficacy, subjective experiences, and existential or psychological transformations that often play a central role in PAT. This prompts a broader question: Should psychedelic research redefine therapeutic success beyond symptom reduction and incorporate measures of psychological, functional, and existential well-being?

### 2.2. Data Sources and Search Strategy

A literature search was conducted in the Web of Science Core Collection to identify clinical studies investigating psilocybin in psychiatric or neuropsychiatric conditions. The search was performed in titles and abstracts, using Boolean logic and combinations of the following keywords linked with “OR”: “psilocybin” OR “clinical trial” OR “study” OR “research”. This approach ensured the inclusion of records in which any of these terms appeared as primary descriptors of the study. We included interventional clinical trials of psilocybin for psychiatric or neuropsychiatric conditions. We excluded preclinical studies, commentaries without primary data, non-English reports, and case reports unless they illustrated specific safety or methodological issues.

We deliberately selected the ten most highly cited clinical trials (Table 1), as these landmark studies have played a central role in shaping contemporary psilocybin research. Since the mid-2010s, influential studies—such as those by Griffiths et al., Carhart-Harris et al., and Ross et al.—have established many of the methodological conventions currently used in PAT research, including dosing paradigms, therapeutic support models, and safety protocols. To reduce temporal bias, we restricted the search to publications from 2015 to 2025, corresponding to the period of the modern clinical revival of psychedelic research. The set of ten papers included two extended analyses [15,16] based on an earlier study by Carhart-Harris and colleagues (2016) [17]. Given that scientific influence is partly shaped by citation impact, these papers were not excluded. Although citation frequency is not a proxy for methodological quality, it does indicate the extent to which specific studies have influenced subsequent research and theoretical development. Examining these studies therefore allows for a critical evaluation of the assumptions, methodological decisions, and limitations structuring the current field.

Additional literature was identified through cross-referencing key studies on psychedelic therapy. Systematic reviews and meta-analyses were also incorporated to provide aggregated assessments of clinical findings. Because psilocybin research is deeply intertwined with historical and cultural contexts, interdisciplinary perspectives were included to support a more comprehensive understanding of both its therapeutic potential and the challenges associated with its medicalisation.

### 2.3. Limitations and Reflexivity

We recognise the relevance of Claude Bernard’s maxim: “Put off your imagination, as you put off your overcoat, when you enter the laboratory. Put it on again, as you put on your overcoat, when you leave.” Nevertheless, as a narrative review, this study does not follow a formal systematic review framework such as PRISMA. The selection of studies was not intended to provide an exhaustive overview of all existing evidence but to enable a focused methodological analysis of influential clinical research. Furthermore, our critique of prevailing epistemic assumptions may limit the depth of discussion regarding specific clinical outcomes in favour of a broader conceptual evaluation of evidence-generation practices within psychedelic research.

## 3. Results

### 3.1. Modern Era of Psilocybin Clinical Trials

While early exploratory psilocybin studies of the mid-20th century were hindered by serious methodological limitations and by policy and regulatory restrictions, contemporary surveys have adopted EBM frameworks, employing RCTs to assess efficacy and safety. Accumulating evidence underscores the therapeutic potential of psilocybin in addressing three main psychiatric conditions: major depressive disorder (MDD), anxiety, and substance dependence. Across various clinical populations, psilocybin administered within structured psychotherapeutic frameworks has been associated with rapid and sustained symptom reduction.

Two landmark RCTs in patients with life-threatening cancer demonstrated that a single dose of psilocybin produced significant and enduring reductions in depression and anxiety, with effects persisting for six months in many cases [18,19]. These findings suggest a potential role for psilocybin in alleviating existential distress in palliative care contexts (Table 2). In TRD, Carhart-Harris et al. conducted an open-label feasibility study revealing substantial symptom reductions following two psilocybin sessions [17]. A six-month follow-up confirmed the durability of the antidepressant effects [15]. In accordance with these findings, a large RCT comparing psilocybin with escitalopram reported comparable efficacy, with psilocybin demonstrating faster onset and fewer side effects [20]. Similarly, Davis reported that two psilocybin sessions led to significant reductions in depression severity among individuals with MDD, with 71% of participants achieving a clinically significant response and 54% reaching remission at four weeks [21]. More recently, Goodwin and colleagues [23] reported a phase II multicentre trial in which a single high dose of synthetic psilocybin was used for TRD, which yielded robust antidepressant effects and functional improvement.

Psilocybin’s therapeutic potential also extends to the treatment of addiction. In a proof-of-concept trial for alcohol use disorder, psilocybin-assisted therapy led to marked reductions in heavy drinking days [22]. Johnson and colleagues demonstrated promising long-term results in tobacco addiction [24]. All 15 participants (100%) attended the 12-month follow-up visit, and 12 participants (80%) completed a long-term follow-up, with a mean interval of 30 months (range: 16–57 months). This represents a high retention rate for a pilot study, suggesting strong participant engagement and good acceptability of the intervention. However, the wide variability in the timing of long-term follow-up indicates that the definition of ‘long-term’ was not uniform (16 months for some participants, 57 months for others). The analysis was based on a continuation of a study originally started by Johnson’s team in 2014, in which 80% of participants remained abstinent at six months, significantly outperforming standard cessation therapies [25].

Notably, the intensity of the acute psychedelic experience appears to predict clinical outcomes. Roseman and colleagues reported that the intensity of the mystical-type experience was predictive of long-term symptom relief, suggesting that the therapeutic mechanism may hinge on experiential rather than purely pharmacological factors [16].

While psilocybin is generally well tolerated, it is crucial to consider potential adverse effects beyond suicidal ideation, which has been reported in both treatment and placebo groups [23]. Other concerns include dysphoria and anxiety during psychedelic sessions, which can be distressing for some patients [18]; the risk of psychotic disorders in vulnerable individuals, particularly those with a history of schizophrenia or bipolar disorder [19]; and potential cognitive impairments following treatment, although current evidence on long-term neurocognitive effects remains limited [18]. These risks necessitate careful patient selection and structured monitoring protocols. Headaches, anxiety, and nausea remain the most common side effects, noted in almost all of the most cited studies.

### 3.2. Methodological Concerns

#### 3.2.1. Selection Bias

Appropriate participant selection remains one of the major methodological limitations in psilocybin research. Much of the literature is derived from studies conducted in Western countries, predominantly involving white adult populations. As such, these findings may not account for pharmacokinetic, cultural, or social disparities among minority groups [26]. A meta-analysis by Metaxa and Clarke, involving 436 participants, revealed that approximately 90% were white, which does not reflect the diversity of real-world clinical settings where psilocybin may be accessible [27]. In 9 of the 10 most cited studies on psilocybin, the white population was the clear majority (Table 3).

The predominance of white participants limits the generalizability of findings to more diverse populations. In light of findings from a longitudinal online survey conducted by Jones et al. demonstrated that certain beneficial effects of psilocybin, such as cognitive flexibility and spiritual well-being, may persist longer in white participants than in non-white participants. Additionally, non-white participants were more likely to experience mystical states and accelerated thinking [28]. Cultural and socioeconomic factors (e.g., healthcare access, social support, educational level) may influence the perception and evaluation of psychedelic effects, an issue recognized by the authors themselves [29]. However, these factors are often overlooked, restricting the generalizability of findings to broader populations. Furthermore, clinical and preclinical studies on psilocybin have shown a disproportionate sex bias [26,30]. The predominance of male participants is particularly problematic in light of the neurobiological interactions between serotonin and estrogen, whose levels fluctuate across the menstrual cycle and throughout a woman’s life [31]. The overrepresentation of men is evident in the 6 studies we analysed.

A significant methodological limitation in psilocybin research is the frequent exclusion of individuals with a history of psychiatric disorders, psychiatrically ill relatives, or those currently prescribed antidepressant medications. This occurred in all the studies analysed. The assumptions thus made about participant selection criteria apply to all the studies reviewed. This may appear counterintuitive given the increasing prevalence of psychiatric disorders in the general population.

However, this practice is predominantly motivated by safety considerations, as conditions such as bipolar disorder and psychoses could be exacerbated by psychedelics. Similarly, the exclusion of individuals on antidepressants is warranted owing to potential pharmacological interactions. Nevertheless, these criteria limit the generalizability of clinical trial outcomes to broader patient populations and fail to address the risks associated with psilocybin use outside controlled environments [32]. On the other hand, the motivation of participants struggling with mental health problems, driven by a desire to improve psychological well-being or experience a psychedelic state, may enhance the placebo effect, complicating the interpretation of results, especially in microdosing studies [33].

**Table 3:** Characteristics of the control and study groups.

Study	Sample Size	Psychiatric Exclusion Criteria	Control Group	Placebo Type
Griffiths et al. (2016) [18]	51 patients with depression/anxiety in terminal illness (26 male, 92% white)	Current medications: psychoactive, anxiolytic, MAO-inhibitors; other than depression psychiatric disorder, relatives with schizophrenia; psychotic; or bipolar disorder	Yes	Active placebo (niacin)
Ross et al. (2016) [19]	29 patients with cancer related depression/anxiety (11 male, 90% white)	Personal/family history of schizophrenia, bipolar disorder, delusional disorder, paranoid disorder, and schizoaffective disorder. substance use disorder, Psychotropic medications for two weeks before and during the study	Yes	Active placebo (niacin)
Carhart-Harris et al. (2016) [17]	12 patients with TRD (6 male, 75% white)	Personal/family history of psychotic disorder or mania, suicide attempt	No	None
Carhart- Harris et al. (2021) [20]	59 patients with moderate-to-severe MDD (39 male, 88% white)	Personal/family history of psychosis, suicide attempt, previous use of escitalopram, previous use of psilocybin, known or suspected pre-existing psychiatric disorder	Yes	Escitalopram
Davis et al. (2021) [21]	24 patients with MDD, (8 male, 92% white)	Current antidepressant medications, history of psychotic disorder, suicide attempt, psychiatric hospitalization	Yes	Nonactive placebo
Bogenschutz et al. (2015) [22]	10 patients with active alcohol dependence (6 male, 30% white non-Hispanic)	History of schizophrenia, bipolar disorder, or suicide in family; drugs- dependence, using hallucinogens more than 10 times	No	None
Goodwin et al. (2022) [23]	233 patients with TRD (112 male, 92% white)	Risk for suicide, history of psychiatric disorder, CBT/ECT therapy, substance abuse, exposure to psychedelics	Yes	Low-dose psilocybin (1 mg)
Carhart-Harris et al. (2018) [15]	20 patients with TRD (14 male, 75% white)	Current or previously psychotic disorder, relatives with a psychotic disorder	No	None
Roseman et al. (2018) [16]	20 patients with TRD (14 male, 75% white)	Current or previously psychotic disorder, suicide attempt, history of mania, relatives with a psychotic disorder	No	None
Johnson et al. (2017) [24]	15 patients with nicotine addiction (10 male, 93% white)	Personal/family history of psychotic or bipolar disorders, drug dependence	No	None

It is worth noting that most studies recruited individuals who were highly motivated and open to experimental therapies. As Johnson’s team (2017) study demonstrated, high participation in follow-up was possible even without financial compensation [24]. Attention should also be given to the small number of participants in clinical trials. Among the studies analysed, only one phase II study had more than 100 participants [23]. This condition makes it impossible to extrapolate the results of the studies to a wider population.

#### 3.2.2. The Issue of Placebo and Blinding

The absence of a placebo effect was found in half of the most cited studies on psilocybin. However, the placebo challenge is further heightened by difficulties in maintaining blinding in studies involving psychedelics. The distinctive effects of psilocybin, such as intense subjective experiences, significantly hinder the ability to “mask” group assignments [34]. For example, in a study by Sloshower, 79% of participants in the placebo group correctly identified their allocation after the first therapeutic session, with this percentage increasing to 80% in the second session [35]. This compromises blinding integrity and may introduce expectation-related bias, as both participants’ and researchers’ expectations can influence treatment outcomes or their interpretation. Moreover, full randomisation becomes difficult to maintain at that point.

Another critical challenge is the lack of an optimal active placebo. While substances such as niacin [19], diphenhydramine [36], and low doses of psilocybin [23] have been employed in various studies, each of these options presents distinct limitations. For example, niacin induces noticeable physical effects, such as skin flushing, which can undermine the integrity of blinding [19]. On the other hand, low doses of psilocybin, although subtler in their effects, may still provoke slight mood or perceptual shifts that could also interfere with the blinding process [36].

Given these challenges, the development of comparative studies is particularly important. The study by Carhart-Harris is among the few that has compared the efficacy of psilocybin with that of an established antidepressant, escitalopram, in the treatment of depression [20]. These research designs provide a more comprehensive assessment of the therapeutic potential of psilocybin [8]. A reanalysis of the results, now using the Bayesian method, conducted by a team led by Nayak with the participation of Carhart-Harris reveals several key mechanisms influencing the efficacy of psilocybin and escitalopram therapies, particularly concerning patient expectations and nonpharmacological therapeutic factors. While psilocybin demonstrated greater efficacy than escitalopram did, the differences did not reach clinical significance, and its efficacy appeared to be unaffected by patient expectations. The findings indicate that in the escitalopram group, higher pretreatment expectations were associated with improved outcomes, which is consistent with prior research on the role of placebo effects in SSRI therapies. In the psilocybin group, the absence of a significant relationship between expectations and therapeutic response might be attributed to blinding challenges, the high suggestibility of participants, or unique pharmacodynamic mechanisms of psilocybin. Furthermore, personality traits such as suggestibility are predictive of psilocybin efficacy, highlighting the role of nonpharmacological factors, such as therapist–patient interactions and environmental influences [36].

The analysis presented in another Bayesian framework, this time by Hsu and colleagues [8] provides insights into the comparative efficacy of psilocybin and escitalopram for treating depression. The study revealed that the placebo response observed in psychedelic trials was lower than that in escitalopram trials. This suggests that the placebo effect in psychedelic research may be overestimated because of issues with blinding. Specifically, high-dose psilocybin was more efficient than placebo in depression trials, but its effect size significantly decreased compared with that of placebo in psychedelic trials. Furthermore, the analysis indicated that high-dose psilocybin was more efficient than escitalopram at both the 10 mg and 20 mg doses, although the size effect of psilocybin was similar to that of escitalopram in major depressive disorder treatment. The efficacy of psilocybin is also context dependent, with the findings of this study suggesting that therapeutic outcomes could stem not only from the pharmacological properties of psilocybin but also from accompanying psychological support. This highlights the complex interaction between pharmacological and psychological factors in the therapeutic potential of psilocybin [8].

On the basis of the results of the comparative analysis, similar conclusions were drawn by Szigeti and colleagues. The researchers also noted a slightly stronger antidepressant effect of psilocybin than escitalopram, although the magnitude of this effect was small (SMD = 0.31). This finding indicates that while psilocybin shows promise, it does not yet surpass the clinical efficacy of established antidepressants. Notably, compared with placebo, no intervention was associated with higher rates of discontinuation or severe adverse events, suggesting a favourable safety profile for psilocybin. These findings underscore the need for further research to refine our understanding of psilocybin’s role in depression treatment, considering its effects in conjunction with psychotherapy and other therapeutic factors [37]. The lack of a proper control group or a satisfactory placebo is particularly problematic when assessing the impact of psychological support on the final study outcomes in the case of PAT. It has taken place in influential trials such as Johnson et al. [24], Carhart-Harris [17,20], and Davis [21].

#### 3.2.3. Set and Setting Limitations in Psilocybin-Assisted Therapy

Research on PAT highlights the critical role of both “set” and “setting” in shaping the outcomes of therapy. “Set” refers to the individual’s mindset and expectations before the experience, whereas “setting” pertains to the environment, such as the presence of a therapist or the physical location of the session [3]. Clinical trials are typically conducted in controlled environments with the support of trained therapists, which maximize positive outcomes and minimize the risk of adverse reactions. However, the artificiality of these controlled settings may limit the applicability of the findings to real-world conditions. For example, mystical-type experiences, which are strongly correlated with positive long-term outcomes, are often more easily achieved in such controlled settings [3]. In contrast, participants may encounter different experiences when the setting or level of psychological support varies [38].

Moreover, the role of support, such as the quality of the therapeutic alliance, remains underexplored in psilocybin research, despite its potential influence on treatment outcomes [38]. In studies conducted by Carhart-Harris [17,20] and Davis [21], patients received individualized psychotherapeutic support, which may have significantly influenced treatment outcomes. PAT is not merely pharmacological; the therapeutic setting, psychological preparation, and integration sessions play a fundamental role in enhancing and sustaining its benefits. The limited exploration of this topic also warrants attention. As Goel et al. suggested [12], future research should investigate the synergistic effects of psychotherapy alongside psilocybin treatment. For example, the authors of the first RCT of psilocybin in the treatment of OCD decided not to validate a psychiatric intervention [39]. Furthermore, the variability of placebo effects in psychedelic studies, influenced by the setting and expectations of participants, underscores the importance of context [12]. Research by Olson and colleagues reveals that even 20 out of 33 placebo participants, when placed in a conducive environment with heightened expectations, may report psychedelic-like experiences [33]. These findings highlight the importance of understanding the complex interaction between pharmacological effects and contextual factors, as both contribute significantly to the therapeutic outcomes of PAT.

#### 3.2.4. Reductionism of Clinical Trials and the Contextuality of Psilocybin Therapy

In the context of PAT, a significant methodological limitation is the reductionist approach often observed in clinical trials. Many studies have prioritized isolating pharmacological effects, potentially underrepresenting contextual determinants of therapeutic response [12]. Carhart-Harris and colleagues reported that the efficacy of therapy is also dependent on the environment, which is not measurable in traditional clinical methods [17]. Typically, these studies are conducted in controlled environments featuring standardized dosing protocols, defined therapeutic structures, and professional therapist support. While such conditions are essential for ensuring the validity of findings regarding the substance’s effects, they fail to capture the complexity of real-world scenarios where psilocybin might be utilized [40].

The reductionism of clinical trials becomes even more problematic when considering the subjective effects of psilocybin and the individual response of patients. These subjective effects often correlate with the therapeutic effect of the drug. Consequently, this limits the generalizability of the results to practical clinical settings [41]. Furthermore, an important question also needs to be asked from an epistemological point of view. The operationalization of subjective “mystical experiences” within quantitative research frameworks remains methodologically challenging. Moreover, while many patients attributed their improvement to mystical-type experiences, the standardization of treatment outcomes remains even more complicated. Although randomised trials are now the dominant framework for evaluating psilocybin, their assumptions and limitations become clearer when viewed against the longer historical trajectory of psychedelic research. Evidence remains mixed: some studies associate “mystical-type” intensity with better outcomes, whereas others find no such relationship when pre-session factors (e.g., relaxation) are considered; expectancy and context can themselves induce psychedelic-like reports under placebo [16,33,42].

### 3.3. History

Psilocybin mushrooms play a significant role in human culture, particularly within spiritual and ritualistic contexts. Archaeological evidence suggests that psilocybin-containing mushrooms are employed in prehistoric rituals, potentially influencing early human cognitive and social development through mechanisms such as neurogenesis and altered perception states [43,44,45,46]. Prehistoric rock art from regions such as Tassili (Algeria), Acacus (Libya), Ennedi (Chad), and Spain, dating back to 9000 BCE, depicts mushroom-like symbols [47,48]. These artefacts suggest early awareness of the psychoactive/mind-altering properties of certain fungi, although their exact role in these societies remains speculative [49].

In Mesoamerica, indigenous groups such as the Aztecs, Zapotecs, and Mixtecs integrated psilocybin mushrooms into religious ceremonies [50]. These mushrooms, referred to as teonanácatl (“flesh of the gods”), facilitated spiritual communion with deities and ancestors. Archaeological findings, including so-called mushroom stones and other ritual artefacts, support the long-standing ceremonial use of psilocybin mushrooms, with traditions traceable to at least 3500 years ago [51]. Only the arrival of Spanish colonizers in the 16th century at Mezzoamerica disrupted indigenous psilocybin practices. The Catholic Church, viewing these practices as heretical, sought to suppress them. Clerics such as Bernardino de Sahagún not only condemned the use of magic mushrooms but also framed indigenous spiritual practices within the moral and theological framework of Catholicism, labelling them as acts of idolatry and sorcery. This translation of indigenous rituals into Catholic terms serves to delegitimize and demonize traditional beliefs [52,53]. Despite these efforts, indigenous use persisted, as evidenced by 17th-century accounts from Francisco Hernández [51,54,55]. However, by the early 20th century, significant misconceptions had emerged, with teonanácatl being incorrectly identified as peyote (Lophophora williamsii) [56,57]. This error was corrected in 1939 when ethnobotanist Richard Schultes confirmed that teonanácatl specifically referred to psilocybin-containing fungi [58,59,60].

The mid-20th century marked the resurgence of interest in psilocybin mushrooms. In the 1950s, R. Gordon Wasson, alongside his wife, documented the ceremonial use of psilocybin mushrooms with Mazatec shaman María Sabina in Oaxaca. Wasson’s 1957 article in Life Magazine brought widespread attention to psilocybin in the West, spurring scientific curiosity [61,62,63,64]. Shortly thereafter, French mycologist Roger Heim identified several Psilocybe species, whereas Swiss chemist Albert Hofmann isolated psilocybin and psilocin from Psilocybe mexicana in 1958. These scientific discoveries prompted further research into the psychoactive properties of psilocybin and its potential therapeutic applications [65].

The 1960s saw a surge in psilocybin research, notably with Harvard psychologist Timothy Leary’s establishment of the Harvard Psilocybin Project [66]. However, the broader cultural context of the 1960s counterculture movement, where psilocybin became popular as a recreational psychedelic, led to increased governmental scrutiny and eventual legal restrictions on psilocybin and similar substances [67].

While the historical and cultural significance of psilocybin is well documented, translating the historical narratives into clinical evidence for its medicinal potential presents several challenges. The first is the lack of historical empirical evidence for medicinal use. Although psilocybin mushrooms have been used for centuries in spiritual and ritual contexts, claims regarding their “mind-healing properties” often lack precise definitions and robust historical substantiation [68]. The term “ritualistically used mushrooms” highlights their ceremonial role rather than any direct and solely medicinal application [55,69]. There is a notable scarcity of primary historical evidence, such as manuscripts or archival records, validating claims of systematic medicinal use [68]. Moreover, the historical psychoactive experiences induced by psilocybin are deeply subjective and socioculturally contextual [70,71,72]. Even if mystical experiences could be neurobiologically reduced to specific brain processes, this reduction fails to explain the qualitative nature of these experiences. The lack of empirical and historical research on the qualitative analysis of these experiences and their descriptions over time in various cultures further complicates this issue. Attempts to standardize such experiences via modern tools such as “Mystical experience questionnaires” or “Awe experience scales” are inherently limited and anachronistic, as they cannot fully capture the depth and cultural specificity of historical psilocybin use and historical language.

Additionally, the interpretive nature of historical studies on “magic mushrooms” poses a significant limitation. Archaeological artefacts and ethnobotanical accounts provide indirect evidence of psilocybin use, but they cannot definitively elucidate the medicinal or therapeutic intentions behind such practices. For example, while teonanácatl was used to facilitate spiritual communion, this does not necessarily imply an understanding of psilocybin as a medicinal compound in the modern clinical sense. Contemporary medical debates require rigorous clinical trials, standardized methodologies, and reproducible results: criteria that historical narratives, by their nature, cannot meet. While historical studies may offer valuable hypotheses regarding psilocybin’s potential, these hypotheses remain speculative without the backing of controlled evidence. Claims that indigenous cultures universally employed psychedelic fungi for therapeutic purposes lack robust historical documentation [68,73]. The changing historical interpretations of psychedelic effects have also shaped the questions pursued in contemporary neurobiology, which now seeks mechanisms for experiences described decades ago.

### 3.4. Neurobiological Modes of Action

While the mechanisms underlying the effects of psilocybin on the central nervous system have been extensively studied, several key aspects remain insufficiently understood and warrant further investigation (Table 4). One of the most pressing gaps concerns the full spectrum of psilocybin interactions with serotonin receptors beyond the well-established 5-HT2A and 5-HT1A subtypes. Although its affinity for other serotonin receptor subtypes, such as 5-HT3, 5-HT6, and 5-HT7, has been documented, the functional significance of these interactions remains unclear [74]. Determining whether these receptors contribute to the psychedelic experience or its therapeutic effects requires further experimental validation. Similarly, while psilocybin has been shown to modulate neurotransmitter levels, including serotonin, dopamine, glutamate, and GABA, the precise role of the cholinergic system in this modulation remains speculative [75]. Evidence suggests that acetylcholine levels may be elevated following psilocybin administration, potentially influencing synaptic plasticity. However, the mechanistic pathways through which psilocybin affects cholinergic signalling require additional research [76].

At the molecular level, while the activation of phospholipase C-β and the formation of 5-HT2A/mGlu2 receptor complexes by psilocybin have been demonstrated, downstream signalling pathways remain incompletely mapped [77]. In particular, the extent to which psilocybin influences the Ras/MAPK and PI3K pathways—both of which are critical to neuroplasticity—remains an open question [78]. Furthermore, recent findings suggest that psilocybin’s neuroplastic effects may, at least partially, be independent of 5-HT2A receptor activation, as ketanserin failed to inhibit these effects in experimental models [79]. These findings suggest that additional receptor-mediated or intracellular mechanisms may contribute to psilocybin’s neuroplastic effects.

**Table 4:** Established knowledge and knowledge gaps in neurobiological mechanisms of action.

Research Area	Scientifically Confirmed	Requires Further Investigation
Metabolism	Psilocybin is metabolized to psilocin in the intestines and liver (Lowe et al., 2021 [74])	-
Serotonin Receptors	High affinity for 5-HT2A, moderate for 5-HT1A, interactions with 5-HT1D and 5-HT2C (Passie et al., 2002 [80])	The full significance of interactions with other serotonin receptors, e.g., 5-HT3, 5-HT6,5-HT7 (Lowe et al., 2021 [74])
Effects on Brain Activity	PET studies confirm strong binding to 5-HT2A in the prefrontal cortex (Halberstad et al., 2011 [81])	Are changes in metabolic activity across brain regions (Gouzoulis-Mayfrank et al., 1999 [82]) fundamental to the psychedelic experience?
Neurotransmitter Modulation	Increases serotonin, dopamine, glutamate, and GABA levels (Wojtas et al., 2022 [75])	Effects on other neurotransmitter systems, such as cholinergic pathways (Wojtas et al., 2023 [76])
Neuroplasticity	Enhances neuroplasticity via TrkB and BDNF signalling (Moliner et al., 2023 [79])	Is neuroplasticity independent of 5-HT2A receptor activation? (Moliner et al., 2023 [79])
Molecular Mechanisms	Activates phospholipase C-β and forms 5-HT2A/mGlu2 complexes (López-Giménez and González-Maeso, 2018 [77])	Influence on Ras/MAPK and PI3K pathways (Yang et al., 2020 [78])
Cerebral Blood Flow (CBF) Regulation	May increase blood flow in specific brain regions (Rieser et al., 2023 [83])	Can relative cerebral blood flow (rCBF) serve as a biomarker for therapeutic efficacy? (Rieser et al., 2023 [83])
Subjective Psychedelic Effects	Correlation between 5-HT2A activation and visual hallucinations (Ling et al., 2022 [84])	The role of individual factors and the microbiome in modulating psilocybin response (Kuypers, 2019 [85])
Antidepressant Effects	Suppresses TNF-α and IL-6, increases BDNF levels (Ling et al., 2022 [84])	Full elucidation of antidepressant mechanisms and long-term efficacy
Functional Connectivity (FC) Modulation	Alters Default Mode Network (DMN) activity and reduces neuronal synchrony (Siegel et al., 2024 [86])	How do FC changes translate into therapeutic outcomes? (Yu et al., 2024 [87])

While neurobiological models help explain how psilocybin may work at a mechanistic level, real-world data are needed to show how these mechanisms translate into meaningful outcomes in everyday clinical practice.

### 3.5. Opportunity of Real-World Studies

RWD and real-world evidence (RWE) have been gaining increasing attention among clinicians [88,89,90,91,92,93]. A pivotal moment for this research approach was in 2016, when the FDA recommended the use of RWD for developing clinical guidelines [94]. In contrast to data obtained from RCTs, RWD data are derived primarily from real-world clinical practice. These data may be both quantitative and qualitative and are often more narrative in nature (Table 5). Real-world studies utilize, among other methods, electronic health records, adverse event monitoring programs, medical claims, family history, mobile health, and patient-reported data [95]. These diverse data can be integrated through pragmatic clinical trials (PCTs), which are considered the most reliable type of real-world research. PCTs allow for the evaluation of the efficacy and safety of a medical intervention in real-world clinical settings rather than in the strictly controlled conditions of clinical trials [96]. Several RWD/RWE studies cited here are based on small samples or online convenience surveys. Estimates are therefore imprecise and vulnerable to selection and expectancy bias; we treat nominal significance as exploratory and hypothesis-generating.

An example of an interesting use of RWE in psychopharmacology is the INVEGA/SUSTENNA study, whose results contributed to the FDA’s decision to expand the therapeutic recommendations for paliperidone palmitate in the treatment of schizoaffective disorders in adults [97].

Despite growing interest, real-world methodology is still underutilized in psilocybin research. However, existing studies have provided valuable insights that classical clinical trials cannot capture. A study conducted by Calder and colleagues aimed to assess whether and how mystical experiences and relaxation are associated with improvements in depressive symptoms in patients who had previously undergone psilocybin or LSD-assisted psychotherapy as part of a limited access program in Switzerland. The intervention effects were compared between a heterogeneous group of patients who had undergone any psychedelic-assisted therapy (*n* = 28) and a healthy control group (*n* = 28) in a double-blind study. The results indicated a significant reduction in depressive symptoms, as measured by the MADRS, with a decrease of 6.2 points (SE = 1.42, *p* < 0.001). However, in a more intriguing finding in the context of seeking valuable evidence, the study did not observe a correlation between “mystical experiences” and the antidepressant effect, contradicting previous findings regarding their crucial role. More importantly, relaxation prior to psychedelic therapy was found to be a stronger predictor of symptom alleviation [42].

Furthermore, patients reported less ‘ego dissolution’ than participants did, which is also considered a predictor of the therapeutic effects of psilocybin. Adverse effects were more frequently reported by patients than by participants, although they were relatively mild (headaches in 35.7% of patients, nausea in 28.6%, and fatigue in 21.4%), resolving within two days and not reaching statistical significance. Notably, a single patient experienced several adverse psychological effects following psilocybin treatment, including anxiety, nonpsychotic hallucinations, cognitive issues, and passive suicidal thoughts, all of which resolved within six days [42]. Understanding contextual factors in PAT is crucial since real-world clinical practice conditions cannot be controlled and are not typically accounted for in RCTs. However, the dynamic environment poses several risks, especially regarding episodically reported serious adverse effects of psilocybin use, such as increased suicidal thoughts [7,98]

In another study utilizing real-world methodology, de la Salle and colleagues analysed the experiences of patients suffering from life-threatening cancer (*n* = 8) after consuming psilocybin mushrooms under naturalistic conditions. To this end, they compared survey results before and two weeks after PAT. The participants described the experience as spiritually and psychologically significant, although it was accompanied by difficult emotions. Two patients considered PAT one of the most challenging experiences of their lives. Five patients reported psilocybin-induced side effects the day after PAT. The most common symptoms included nausea and vomiting (4/5), crying (3/5), headache (3/5), and sweating or chills (3/5). One patient experienced a significant decrease in mood after the psilocybin experience. Importantly, there were almost no changes in attitudes towards death (e.g., fear or avoidance on the DAP-R scale) or medical assistance in dying patients. The PHQ-9 depression scale score decreased from 6.4 to 3.8, indicating moderate but significant improvement (SE mean difference = 1.6, *p* = 0.1). However, the initial value of 6.4 should be considered a mild level of depressive symptoms. A significant reduction in depressive symptoms was noted on the ESAS-R scale (from 5.0 to 2.9; mean difference SE = 0.7; *p* = 0.02), although this tool is not specific for assessing depression but rather overall health in palliative care patients. Scholars have also reported a significant reduction in anxiety symptoms (GAD-7 scale) and an increase in overall quality of life (MQoL-R) [99].

The real-world context of psilocybin research is also enriched by online studies on recreational users of psychedelics. In 2019, Anderson et al. conducted a survey on a sample of 278 anonymous psilocybin users (*n* = 50), LSD users (*n* = 195), or both substances (*n* = 33) in microdoses. The analysis focused on the benefits and challenges associated with microdosing. Among the top three reported benefits were mood improvement (26.6%), improved focus (14.8%), and increased creativity (12.9%). The most reported drawbacks included illegality (29.5%), psychological discomfort (18.0%), and decreased focus (8.8%) [100]. Because the original publication did not report confidence intervals for these proportions, the findings should be interpreted as exploratory and descriptive, rather than as precise estimates of effect magnitude.

Somewhat different data regarding the challenging experiences associated with psilocybin use were provided by extensive online surveys conducted by Barrett et al. with sample sizes of *n* = 1993 (95% CI: 0.054–0.059) and *n* = 981 (95% CI: 0.053–0.058). Through multivariate analysis, the researchers demonstrated a correlation between the increased intensity of these challenging experiences and neurotic personality traits [70]. Notably, in the context of the interaction between psilocybin and the most common antidepressants, selective serotonin reuptake inhibitors (SSRIs) have been studied by Sakai. Scholars identified 443 posts on the Reddit platform regarding the coadministration of psilocybin mushrooms and SSRIs. The analysis revealed that 54% of the posts described a reduction in intense psilocybin experiences, 39% reported no changes in the experience, and 8% had negative psychological or physical interactions [101].

## 4. Discussion

Taken together, historical perspectives explain why context matters; neurobiology clarifies how psilocybin may alter brain function; RCTs estimate efficacy under strict controls; RWD/RWE test effectiveness, heterogeneity and durability in practice. A coherent evaluation of PAT therefore requires integrating these strands rather than privileging any single method.

When evaluating PAT or psilocybin pharmacotherapy more broadly, traditional RCTs may not fully capture the complexity of the intervention. A Cochrane review by Schipper et al. [102], which assessed three pivotal RCTs of psychedelic-assisted therapy—Gasser et al., Griffiths et al., and Ross et al. [18,19,103]—rated the certainty of evidence for outcomes including anxiety, depression, existential distress, quality of life, and spirituality as low or very low according to the GRADE framework. These ratings primarily reflected small sample sizes, short follow-up periods, and methodological constraints inherent to the available trials. Similar limitations were noted in a scoping review by Goel and colleagues [12], who emphasized the difficulty of drawing long-term conclusions from current datasets. Despite more than two decades of renewed investigation and over 130 registered clinical studies, psilocybin has not yet received broad regulatory approval for psychiatric indications. As discussed by Norring and Spigarelli [1], this situation likely reflects not only evidentiary limitations but also regulatory caution, challenges in standardizing psychotherapeutic components, and uncertainties regarding long-term safety monitoring. The distinctiveness of PAT lies in the interdependence of pharmacological effects and experiential context. Therapeutic outcomes appear to emerge from an interaction between 5-HT2A-mediated neurobiological processes, structured psychological support, and the patient’s subjective state during the session [33]. This interdependence complicates attempts to isolate a single causal pathway within conventional RCT designs. While reductionist approaches may clarify certain mechanisms, they risk overlooking how the intervention functions as delivered in practice.

High ratings of “personal meaning” or “spiritual significance,” frequently reported in psychedelic trials, may contribute to observed clinical improvement by strengthening motivation, enhancing goal coherence, and reshaping aspects of patient identity. Experimental work on expectancy and contextual modulation—including placebo-controlled psychedelic paradigms—demonstrates that subjective experience can be substantially shaped by environmental and interpersonal factors [33]. Without adequately controlled comparators, it remains difficult to disentangle the respective contributions of set and setting, expectancy effects, therapeutic alliance, and pharmacological action.

An interdisciplinary research strategy may therefore be warranted. Historical analyses question the widespread assumption that hallucinogenic mushrooms were systematically used for medicinal purposes in premodern societies, highlighting instead predominantly ritual and ceremonial contexts. At the same time, contemporary neurobiological research underscores both progress and uncertainty. Although receptor-level mechanisms and neuroplastic pathways have been increasingly characterized, significant knowledge gaps remain—particularly regarding long-term effects, including potential genetic, reproductive, and biomarker-related implications [104]. Real-world investigations further complicate the picture by demonstrating variability in patient populations, therapeutic settings, and safety outcomes under less controlled conditions.

High-quality RWD, if methodologically integrated with randomized designs, may help address some of the limitations inherent in traditional trial paradigms. Observational cohorts and pragmatic clinical trials can broaden inclusion criteria, capture longer follow-up intervals, and provide insight into drug interactions and heterogeneous response patterns. Such approaches cannot replace RCTs, but they may strengthen external validity and clarify how psilocybin-based interventions perform beyond highly selected research environments. At the same time, expanding access to psilocybin therapies in advance of robust confirmatory evidence raises broader methodological concerns. Widespread implementation based on limited-certainty evidence may challenge established hierarchies within EBM, particularly the centrality of randomisation, blinding, control groups, and appropriate placebo conditions embedded within GRADE assessments. Consensus statements in psychiatry continue to emphasize the necessity of placebo-controlled designs and the expectation that novel agents demonstrate efficacy at least comparable to existing therapies [105]. These standards underpin regulatory reliability and protect against premature therapeutic adoption.

The emerging field of psychedelic research therefore occupies a transitional evidentiary position. Signals suggesting therapeutic potential are increasingly difficult to dismiss. Yet confidence intervals surrounding these effects remain wide. Strengthening the evidence base will likely require larger multicentre trials with extended follow-up, improved measurement of expectancy effects, more rigorous blinding strategies, standardized psychotherapeutic protocols, and structured integration of real-world registries. Only through such cumulative refinement can psilocybin-assisted interventions be evaluated within a framework that preserves methodological rigor while accommodating the hybrid nature of the therapy.

## 5. Conclusions

Findings from RCTs position psilocybin as a promising candidate for novel psychiatric interventions. Although substantial progress has been made in characterizing the pharmacological and neurophysiological effects of psilocybin, critical knowledge gaps persist. Addressing these uncertainties will require interdisciplinary approaches that combine molecular neuroscience, neuroimaging, and clinical research to comprehensively delineate the therapeutic mechanisms of psilocybin. Consequently, while the historical and cultural contexts of psilocybin use offer insights into human interactions with psychoactive substances, these narratives are of limited utility in contemporary medical debates about psilocybin’s therapeutic potential. Historical studies cannot substitute for the methodological rigor required in current EBM research, but by reframing indigenous practices through a clinical perspective, researchers can gain valuable insights that inform contemporary therapeutic approaches in PAT. One of the most important features of traditional psilocybin use was the ceremonial setting in which the substance was consumed. These rituals were carefully orchestrated, often led by shamans, and imbued with spiritual meaning. This structured environment—set and setting—was integral to shaping the psychological and emotional outcomes of the experience. In the clinical setting, this principle translates into the use of curated therapeutic environments. Today, therapists adopt roles akin to guides or facilitators, preparing patients through psychotherapy, managing expectations, and ensuring that the setting is conducive to emotional safety and introspection. Just as the ritualistic framework once mitigated fear and enhanced healing, a thoughtfully designed clinical environment has been shown to reduce anxiety and optimize treatment outcomes in psilocybin therapy.

Further large-scale, multicentre studies with extended follow-up are required to establish long-term efficacy and safety. These studies should be conducted by independent centres on a larger, diversified group of participants and patients over a longer period of time to allow extrapolation of results to a large population. Furthermore, the results should be related to the efficacy and safety of known and used psychiatric drugs. However, the application of real-world studies may ensure a balance between real-world conditions and methodological rigor. These features may enhance external validity while preserving methodological rigor. The integration of RWD not only enriches our understanding of psilocybin’s therapeutic potential but also bridges critical gaps left by traditional methodologies. By capturing diverse patient experiences and long-term outcomes, RWD serves as an indispensable complement to randomized trials, paving the way for a holistic approach to evaluating psilocybin’s effectiveness and safety in real-world settings. Future work should integrate larger randomised trials with prospective RWD registries and pragmatic designs, harmonise psychotherapeutic protocols, and pre-specify expectancy and context measures, thereby improving both internal and external validity [96,97].

## Figures and Tables

**Table 1:** Citation Count of Selected Psilocybin Research Studies.

#	Study Title	First Author	Year	Journal	Citations
1.	Psilocybin produces substantial and sustained decreases in depression and anxiety in patients with life-threatening cancer: A randomized double-blind trial	Griffiths, RR [18]	2016	Journal of Psychopharmacology	1483
2.	Rapid and sustained symptom reduction following psilocybin treatment for anxiety and depression in patients with life-threatening cancer: a randomized controlled trial	Ross, S [19]	2016	Journal of Psychopharmacology	1183
3.	Psilocybin with psychological support for treatment-resistant depression: an open-label feasibility study	Carhart- Harris, RL [17]	2016	Lancet Psychiatry	1014
4.	Trial of Psilocybin versus Escitalopram for Depression	Carhart- Harris, R [20]	2021	New England Journal of Medicine	956
5.	Effects of Psilocybin-Assisted Therapy on Major Depressive Disorder: A Randomized Clinical Trial	Davis, AK [21]	2021	JAMA Psychiatry	927
6.	Psilocybin-assisted treatment for alcohol dependence: A proof-of-concept study	Bogenschutz, MP [22]	2015	Journal of Psychopharmacology	880
7.	Single-Dose Psilocybin for a Treatment-Resistant Episode of Major Depression	Goodwin, GM [23]	2022	New England Journal of Medicine	711
8.	Psilocybin with psychological support for treatment-resistant depression: six-month follow-up	Carhart-Harris, RL [15]	2018	Psychopharmacology	689
9.	Quality of Acute Psychedelic Experience Predicts Therapeutic Efficacy of Psilocybin for Treatment-Resistant Depression	Roseman, L [16]	2018	Frontiers in Pharmacology	556
10.	Long-term follow-up of psilocybin-facilitated smoking cessation	Johnson, MW [24]	2017	American Journal of Drug and Alcohol Abuse	467

Note: Number of citations is current as of 9 January 2026. The citation counts are based on data available at that time and may change over time as new citations are added.

**Table 2:** Methodology and Outcomes of Most Cited Psilocybin Trials.

Study	Methodology	Primary Outcome	Adverse Events
Griffiths et al. (2016) [18]	Randomized, double-blind trial	80% reduction in depression/loneliness symptoms at 6 months	Nausea (10%), dizziness (8%), transient paranoia (5%)
Ross et al. (2016) [19]	Randomized, double-blind, crossover trial	83% anti-depressant response rate in the psilocybin first group vs. 14% in the niacin first group and 58% anxiolytic response in the psilocybin first group compared to 14% in the niacin first group at 7 weeks	Elevation in BP and HR (76%), headaches (28%), transient anxiety (17%), nausea (14%)
Carhart- Harris et al. (2016) [17]	Open-label feasibility study	67% reduction in depression after 1 week, 42% maintained improvement at 3 months	Anxiety (25%), transient headaches (17%), nausea (8%)
Carhart- Harris et al. (2021) [20]	Phase 2, randomized, double-blind clinical trial	8.0 pts. decrease on QIDS-SR16 in psilocybin group and 6.0 pts. decrease on QIDS-SR16 in escitalopram group after 6 weeks	73% related adverse events in psilocybin group and 79% in escitalopram group
Davis et al. (2021) [21]	Randomized, waiting list–controlled clinical trial	71% response rate in psilocybin group vs. 48% in placebo at 4 weeks	Anxiety (12%), transient hypertension (8%), nausea (4%)
Bogenschutz et al. (2015) [22]	Proof-of-concept study	26% decrease in heavy drinking days and 27% decrease in total drinking days	Mild headaches (50%), nausea (10%), insomnia (10%)
Goodwin et al. (2022) [23]	Phase 2, randomized double-blind clinical trial	12.0 pts. reduction (25 mg psilocybin group) and 7.9 pts. reduction (10 mg) on the MADRS scale after 3 weeks	Headache (24%), nausea (22%), dizziness and fatigue (6%) in high dose psilocybin group
Carhart- Harris et al. (2018) [15]	Open-label feasibility study	Decrease on QIDS-SR16 at maximum effect size at 5 weeks (−9.2 pts.)	Minutes-long anxiety (75%), headache (40%), transient nausea (25%)
Roseman et al. (2018) [16]	Open-label feasibility study	Significant interactions of Time-OBN, and Time-DED within-subjects effect and the within-subjects contrasts at each time point (*p* < 0.05)	Minutes-long anxiety (75%), headache (40%), transient nausea (25%)
Johnson et al. (2017) [24]	Open-label pilot study	60% met the criteria for abstinence in long term.	Mild headache (67%), subjective psychological side effects (40%)

**Table 5:** Comparison of RCTs in Psilocybin and Potential of Real-World Studies.

Criteria	Method	
	RCT	RWE/RWD
Aim of study	Efficacy	Effectiveness
Population	Selected	Heterogeneous
Study group	Small	Large
Data type	Short-term, reduced	Long-term, comprehensive
Cost	High	Moderate
Conditions	Controlled	Natural
Methodological rigour	High	Moderate

## Data Availability

No data was generated during the study.

## Abbreviations

The following abbreviations are used in this manuscript:

EBM	Evidence-Based Medicine
PAT	Psilocybin-Assisted Therapy
RCT	Randomized Controlled Trials
RWD	Real-World Data
RWE	Real-World Evidence
